# Demyelinating disease and anti-N-methyl-D-aspartate receptor immunoglobulin G antibodies: a case report

**DOI:** 10.1186/1756-0500-7-948

**Published:** 2014-12-23

**Authors:** Anne Waschbisch, Bernd Kallmünzer, Stefan Schwab, Philipp Gölitz, Angela Vincent, De-Hyung Lee, Ralf A Linker

**Affiliations:** Department of Neurology, Friedrich-Alexander University of Erlangen, Schwabachanlage 6, 91054 Erlangen, Germany; Department of Neuroradiology, Friedrich-Alexander University of Erlangen, Schwabachanlage 6, 91054 Erlangen, Germany; Nuffield Department of Clinical Neurosciences, University of Oxford, John Radcliffe Hospital, Oxford, OX3 9DU UK

**Keywords:** Multiple Sclerosis, NMDA receptor antibody, Encephalitis, Demyelinating disease, Anti-neuronal antibodies, Neuroinflammation

## Abstract

**Background:**

Anti–N-methyl-D-aspartate receptor immunoglobulin G antibodies directed against the GluN1 subunit are considered highly specific for anti-N-methyl-D-aspartate receptor encephalitis, a severe clinical syndrome characterized by seizures, psychiatric symptoms, orofacial dyskinesia and autonomic dysfunction.

**Case presentation:**

Here we report a 33 year old Caucasian male patient with clinically definite multiple sclerosis who was found to be positive for anti-N-methyl-D-aspartate receptor antibodies. Rituximab therapy was initiated. On the 18 months follow-up visit the patient was found to be clinically stable, without typical signs of anti-N-methyl-D-aspartate receptor encephalitis.

**Conclusion:**

Our findings add to the growing evidence for a possible association between anti-N-methyl-D-aspartate receptor encephalitis and demyelinating diseases.

## Background

Patients with immunoglobulin (Ig) G antibodies to the GluN1 subunit of the N-methyl-D-aspartate receptor (NMDAR) develop a characteristic clinical syndrome that was termed anti-NMDA receptor encephalitis [1]. These patients may present with acute behavioural changes, dyskinesia or seizures followed by a decrease in consciousness, psychosis, central hypoventilation and autonomic dysregulation. Here we report a male patient who had signs and symptoms highly suggestive of multiple sclerosis (MS) and met the McDonald criteria [2] and yet was found to be seropositive for NMDAR IgG antibodies. Given the high specificity of this antibody, the question arises whether NMDAR encephalitis may mimic MS or whether NMDAR IgG is a coincidental finding either without clinical significance or indicative of a clinically silent (prodromal) state of the disease. Nowadays diagnostic opportunities may challenge the clinician’s appraisal and impose once more the question: treat the patient or his lab results – or both?

## Case presentation

This 33 year-old Caucasian male patient presented to our emergency department with mild left-sided hemiparesis that had developed within days. Six years prior, he had been treated for subacute-onset diplopia at another hospital. At that time, MRI demonstrated several demyelinating periventricular lesions with one gadolinium enhancing lesion. Cerebrospinal fluid (CSF) analysis revealed oligoclonal bands. Following a glucocorticoid pulse therapy complete remission was reached. He was lost to follow up and no further MRI scans or neurological examinations were performed since he remained asymptomatic and felt to be in good health.

**Figure 1:**
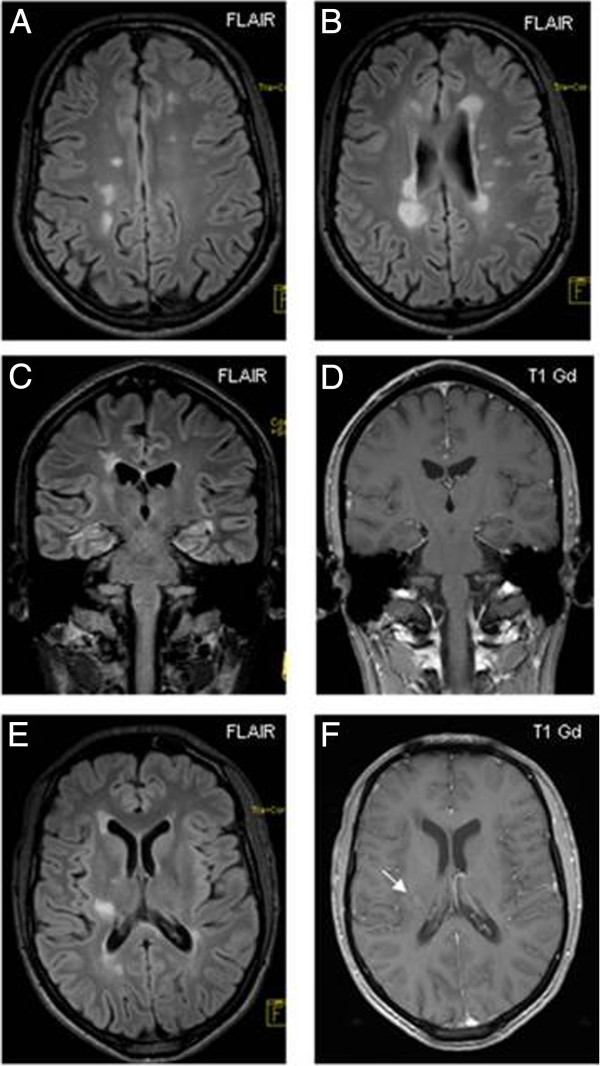
**Magnetic resonance imaging.** MR FLAIR imaging demonstrates multiple hyperintense lesions suggestive of multiple sclerosis **(A-C**, **E)**. One of the lesions (arrow) was found to be Gadolinium (Gd) enhancing on T1 scans **(D**, **F)**.

Cranial MRI on admission demonstrated multiple T2 hyperintense lesions (periventricular, juxtacortical, infratentorial), one of them with gadolinium enhancement (Figure 1). Upon comparison to the MRI six years before, an obvious increase of the lesion load was noted. Visually evoked potentials showed prolonged latencies in both eyes. CSF analysis revealed a borderline pleocytosis (5 cells/μl, 95% lymphocytes). The IgG index (1.3) indicated intrathecal IgG synthesis. A polyspecific intrathecal immunoglobulin synthesis against rubella, varicella and herpes simplex virus but not measles (MRZH reaction) was detected. Oligoclonal bands were found to be positive in serum and cerebrospinal fluid with additional bands in the CSF. Blood work and urine testing were unremarkable without evidence of chronic infection (syphilis, borreliosis, HIV, HBV and HCV), vitamin B12 deficiency or systemic autoimmune disease (ANA, ENA, ANCA, RF, dsDNA and anti-phospholipid antibodies, ACE). The patient was diagnosed with relapsing remitting MS according to the revised McDonald criteria [2] and received a glucocorticoid pulse (5 days, methylprednisolone 1 g/day i.v.) followed by complete recovery. 3 weeks later he returned complaining of new-onset paroxysmal tingling and cramping in his left hand and was found to have tonic spasms that responded well to another course of glucocorticoids and intermittent low dose carbamazepine therapy. Given the higher incidence of tonic spasms in neuromyelitis optica compared to MS [3] we decided to test for aquaporin-4 (AQ4) autoantibodies before initiation of immunomodulatory therapy. Serum samples were sent to an accredited commercial laboratory with long-standing experience (Stöcker Laboratories, Euroimmun AG, Lübeck) that employs a biochip to test for AQ4 autoantibodies in a cell based assay. This biochip consists of a mosaic of fixed human embryonal kidney 239 cells each expressing different recombinant antigens (AQ4, Glu1 NMDAR, AMPAR, GABA-bR, LGI, CASPR2, Amphiphysin, GAD, Hu, Ri, Yo, Tr, MAG, Myelin, Ma/Ta, Glycine receptor) in addition to frozen sections of rat hippocampus and cerebellum. The patient turned out to be AQ4 autoantibody negative but surprisingly IgG directed against the NR1 subunit of the NMDAR (titre 1:100) was detected and confirmed by a typical staining pattern on rat brain. Control testing with an independent serum sample yielded the same result. A third serum sample was sent to a second laboratory (A. Vincent, Imunology Laboratory, Churchill Hospital, Oxford, GB) and confirmed the results and titres.

Further diagnostic tests were performed: Electroencephalography showed a normal alpha-rhythm without evidence for epileptic activity or slowing. Neuropsychological evaluation (Wechsler Memory Scale Revised Edition; Digit span forward/backward; Spatial span backward/forward; Logical memory I, Go/Nogo, Divided attention version I/auditive-visual) demonstrated normal cognitive functions. A psychiatric exploration as well as thorax/adomen CT and spinal cord MRI were unremarkable. A B cell depleting therapy with rituximab was started and well tolerated. A complete B cell depletion was confirmed by immune cell phenotyping. Since then (18 months) the patient has been monitored at short intervals and repeatedly been assessed for seizures, cognitive decline or behavioral changes as well as symptoms of MS relapse but was considered clinically stable. In addition MRI demonstrated paraclinical stability 12 months after the onset of symptoms. NMDAR Ab testing demonstrated persistence at an unchanged titre of 1:100.

## Discussion

Here we describe a patient with clinically definite MS and the presence of NMADR serum antibodies. He was carefully evaluated for seizures, dyskinesia, psychiatric or neuropsychological symptoms which were all negative. Thus, the clinical line-up found was best compatible with relapsing remitting MS and not considered indicative of NMDAR encephalitis.

In contrast to IgA and IgM antibodies directed against the NMDA receptor, NMDAR IgG serum antibodies are in general considered highly specific for NMDAR encephalitis. However, depending on the diagnostic test, false positive IgG serum results have been reported in up to 3% of healthy controls [4, 5]. In our patient, a positive cell based assay [6], was confirmed by a typical staining pattern on rat brain tissue, and in another laboratory. According to previously published studies CSF adds to the sensitivity [7] and the specificity [4] of NMDAR antibody tests. Unfortunately CSF was not available to confirm intrathecal synthesis and the patient declined to undergo a second lumbar puncture which limits the significance of our findings. Also unfortunately, we were unable to obtain high quality images of the IHC/IF stainings done by Labor Stöcker which would have enhanced our report.

Monosymptomatic disease courses of NMDAR encephalitis (“forms frustes”) have been reported. Accordingly, NMDAR IgG Ab were recently described at low frequency (1.7%) in a cohort of epilepsy patients who did not display other features of encephalitis [8]. Similarily, patients with isolated psychiatric symptoms at onset or during relapse were reported [9]. In addition persistence of NMDAR IgG after an initial episode of encephalitis as well as relapses following long clinically silent periods have been reported [10]. Therefore, NMDAR serum IgG may as well precede the clinical onset of NMDAR encephalitis.

A correlation of serum titers with disease activity is a subject of ongoing debate, but a recent study show that higher antibody titers are associated with poor outcome or the presence of teratoma [7]. Our patient had a rather low serum titer of 1:100 (normal <1:10) and tumor screening remained negative.

Anecdotal evidence points towards a possible association of NMDAR encephalitis with demyelinating disease: NMDAR IgG have been reported in children with demyelinating syndromes (ADEM, ON) [11] and extensive myelitis [12]. In addition two cases of patients that initially presented with typical symptoms of NMDAR encephalitis and developed a disease closely mimicking neuromyelitis optica have been reported [13, 14] and a range of demyelinating conditions were reported in case series in adults and children [15, 16].

In contrast a study testing NMDAR IgG antibodies in a small cohort of MS patients as a disease control failed to detect the autoantibody [6]. Herpes encephalitis has been reported as a possible trigger of NMDA receptor antibody formation and consecutive encephalitis [17–19]. The mechanisms remain unknown. However, both molecular mimicry and breakdown of immunologic tolerance towards the NMDAR released by neuronal damage in an inflamed environment have been discussed [17, 18]. Whether tissue damage and inflammation in MS may also trigger the formation of this antibody remains speculative.

## Conclusion

The growing availability of chip technologies that perform simultaneous testing of multiple antibodies independent of the clinical syndrome and with limited confirmatory tests poses the risk of false-positive results and challenges clinicians and laboratory physicians alike who have to decide whether these autoantibodies may indicate an evolving autoimmune process or represent a clinical non-significant epiphenomenon. Further research is warranted to clarify a possible link between demyelinating diseases and NMDAR encephalitis.

In general, diagnosis of any autoimmune disease necessitates the combination of a compatible clinical picture together with conformed laboratory findings and treatment should not be based on laboratory findings alone.

## Consent

Written informed consent was obtained from the patient for publication of this Case Report and any accompanying images. A copy of the written consent is available for review by the Editor-in-Chief of this journal.

## Abbreviations

ACE: Angiotensin-converting-enzyme
ANA: Antinuclear antibodies
ENA: Extractable nuclear antigens
HBV: Hepatitis B virus
HCV: Hepatitis C virus
IgG: Immunoglobulin G
MS: Multiple sclerosis
NMDAR: N-methyl-D-aspartate receptor
RF: Rheumatoid factor.
